# The COVID-19 Pandemic and Posttraumatic Stress Disorder: Emotional Impact on Healthcare Professions

**DOI:** 10.3389/fpsyt.2022.832843

**Published:** 2022-04-01

**Authors:** Concetta De Pasquale, Daniela Conti, Carmela Dinaro, Rosa Alessia D'Antoni, Elena La Delfa, Santo Di Nuovo

**Affiliations:** ^1^Vascular Surgery and Organ Transplant Unit, University Hospital of Catania, Catania, Italy; ^2^Department of Educational Sciences, University of Catania, Catania, Italy; ^3^Department of Humanities, University of Catania, Catania, Italy; ^4^Drug Addiction Health Service, SER.T-ASP3, Catania, Italy

**Keywords:** COVID-19 pandemic, Fear of COVID-19 scale, posttraumatic stress disorder (PTSD), emotional disorders, burnout syndrome (BS), healthcare workers

## Abstract

The COVID-19 pandemic, which began in March 2020, has resulted in the deaths of hundreds of thousands of people around the world in just a few months, putting at great risk the commitment of healthcare workers unprepared to manage a worldwide phenomenon at great risk. In the early stages especially, medical staff had to deal with the pandemic at the expense of their physical and mental health, putting them particularly at risk for experiencing posttraumatic stress disorder (PTSD). The study aims to analyze the psychopathological aspects associated with PTSD, focusing on the emotional impact caused by the COVID-19 pandemic on healthcare professionals compared with a control group. The sample analyzed over 2 months, from March to May 2021, included 214 participants into two groups, i.e., healthcare professionals (*N* = 107) and a control group (*N* = 107). The online assessment instrument used consisted of an anonymous questionnaire, assembled *ad hoc* with demographic information and different standardized assessment scales (e.g., Fear of COVID-19 scale, Profile of Mood States, and Maslach Burnout Inventory-Human Services Survey), while a further section of the survey used the DSM-5 criteria to investigate Posttraumatic stress disorder (e.g., COVID-19—PTSD). The results reported that healthcare professionals had a consistent perception of stress (mean = 26.18, *SD* = 14.60), but not at a level significantly higher than other categories of workers (mean = 25.75, *SD* = 14.65; *t* = 0.20, *p* = 0.84). However, they showed less emotional disturbance than the control sample, better anxiety management skills, and lower levels of depressive disorder and mental confusion. Specifically, the healthcare professionals showed a condition of emotional exhaustion (T = 0.64, D = 0.74, A = 0.62, S = 0.75, C = 0.64) and depersonalization (T = 0.41, D = 0.52, A = 0.49, S = 0.60, C = 0.40), which is common in the burnout syndrome. In conclusion, the results obtained are useful in understanding the determinants of the emotional involvement of healthcare professions and the risk of burnout syndrome and, therefore, for planning activities and support paths for these workers who are particularly at risk during prolonged and pervasive crises, such as the pandemic.

## Introduction

On March 11, 2020, the WHO declared the coronavirus pandemic and SARS-CoV-2 as the causative agent of the COVID-19 respiratory syndrome. The COVID-19 pandemic has attracted worldwide attention for its rapid diffusion: In fact, the highly contagious nature of SARS-CoV-2 has been a major reason for the increasing number of deaths due to COVID-19. Social distancing, confinement, and quarantine were adopted by many countries to contain the diffusion of the infection (1). The literature on the psychological effects of quarantine indicates that the perception of the traumatic event can concern both the fear of contracting the virus and the measures adopted to counter the spread of infection (2).

These extreme measures taken to limit the spread of COVID-19, as well as the fear of contracting the virus, have impacted on people's lifestyles, generating high levels of psychological distress, anxiety, and mood alterations (3, 4). Consequently, they can represent risk factors for many mental health issues and can potentially generate posttraumatic stress disorder (PTSD) symptoms (1, 5).

The DSM-5 (6) indicates that “experiencing repeated or extreme exposure to aversive details of the traumatic event(s)” can be considered as potentially traumatic events. The clinical characteristics required by DSM-5 to define the diagnosis of PTSD provide for the fulfillment of Criterion A, concerning exposure to trauma; moreover, in PTSD, the trauma resurfaces in an intrusive, invasive way, in the subject's memories through flashbacks, vivid images and nightmares, associating with avoidance behaviors of thoughts, places, objects, and situations that recall the traumatic event, with symptoms of affective dulling, negative alterations in cognition and mood, as well as persistent symptoms of increased arousal (Criteria B, C, D, and E of the DSM-5). In addition, a further Criterion F is defined, concerning the significant impairment of social function, work, or other important areas for the individual. In accordance with the criteria expressed in the DSM-5, several studies have been conducted relating to posttraumatic stress disorder.

Although most of the epidemiological studies on PTSD have been conducted in the United States [e.g., (7)], there are some concerning the general European population (8), in particular, the Italian one (9, 10).

An important study was conducted by the European Study of the Epidemiology of Mental Disorders, which analyzed the population of Western Europe, within the WHO World Mental Health Survey Initiative (ESEMeD-WMH); this is a worldwide epidemiological study aimed at estimating the prevalence of PTSD and its association with various traumatic events in the adult population (11). There is evidence of a gender difference in PTSD, with females being more at risk of developing the condition than males (12). Regarding age, some authors have reported that exposure to trauma decreases over the years (13), and other studies show that young age is globally a risk factor for the development of PTSD (14). The pandemic outbreak of an unrecognized infection, such as COVID-19, could be defined as a traumatic experience for its acute and chronic implications at individual and community levels (1). Specifically, the healthcare workers in emergency care settings are particularly at risk of PTSD because of the highly stressful work-related situations they are exposed to, which include: management of critical medical situations, caring for severely traumatized people, frequent witnessing of death and trauma, operating in crowded settings, and interrupted circadian rhythms due to shift work (15). Consequently, investigating the psychological impact of the COVID-19 pandemic on healthcare workers including physicians and nurses has become increasingly important (15, 16).

### Aims and Hypotheses

Currently, given the enormous burden of distress and potentially traumatic events experienced by people who work in healthcare, it is important to document the prevalence of mental health problems in this population group (17). In this framework, the purpose of the current study was to investigate the emotional impact and the prevalence of self-reported PTSD symptoms caused by the COVID-19 pandemic by comparing Italian healthcare professionals to a control group of the general population. Specifically, the main hypothesis was to compare the perception of stress between the two groups and its psychological and clinical effects on the lives of participants.

## Materials and Methods

### Participants and Procedure

The inclusion criteria for volunteers were (i) Italian-speaking citizens, (ii) at least 20 years old, (iii) with at least 13 years of education, and (iv) carrying out work in the healthcare sector or not employed (for the control group). Respondents who did not complete the questionnaire on demographic characteristics and who reported psychological distress before the pandemic were preliminarily excluded. We randomly selected, among the 371 initial respondents, those suitable to balance the groups of health workers and control workers by number, age, and gender. The final sample analyzed in the study included 214 participants into two groups, i.e., healthcare workers (*N* = 107) and control group (*N* = 107). Specifically, each group was composed of 29 men (27%) and 78 women (73%), all aged between 20 and 60 (M = 26.75, *SD* = 3.86). Also, age ranges were matched in the two groups: ages 20–30, *n* = 23; ages 31–40, *n* = 29; ages 41–50, *n* = 24; age > 51, *n* = 31. However, considering the small number of the general sample, it is only representative of the population investigated. Specifically, the two groups had the following characteristics: the healthcare workers (HCWs) included nurses (*N* = 63), doctors (*N* = 19), healthcare assistants (*N* = 9), and medical and nursing students trainees in hospitals (*N* = 14).

The hospitals involved in the study were the University Hospital “Policlinico—San Marco” and the Drug Addiction Health Service, SER.T-ASP3, of Catania. The control group included employed, self-employed, casual employees, housewives, and not employed.

The volunteer participants were informed of the research via email and subsequently gave online written informed consent and answered the questionnaire anonymously. The administration time of the instrument used was ~20–30 min. In the research presentation platform, it was reported that the volunteer participant could leave the completion of the questionnaire at any time of administration.

Data collection occurred from March 14, 2021 to May 30, 2021, namely, 1 year after the onset of the pandemic. In Italy, during this time, the first dose of vaccine and medical treatments were available for the population. Also, the data were collected in aggregate form, and individual users were not identified.

This study was conducted according to the Declaration of Helsinki and was approved by the Ethics Committee of the Department of Educational Sciences at the University of Catania (Italy), which guarantees the confidentiality and anonymity.

The research design was of a correlational type as the objective of the study was to investigate the relationship between the variables used without the researcher controlling or manipulating any of them.

### Measures

The online instrument with 112 items consisted of an anonymous questionnaire, assembled *ad hoc* including demographic information and different standardized assessment scales.

The first part of the questionnaire was on sociodemographic parameters (e.g., gender, age and profession), while the second part consisted of standardized scales, i.e., the Fear of COVID-19 scale (FCV19S) (18), the COVID-19—Post Traumatic Stress Disorder (COVID-19-PTSD) (1, 19), the Profile of Mood States (POMS) (20), and the Maslach Burnout Inventory—Human Services Survey (MBI-HSS) (21).

The final part of the questionnaire was a debriefing. The volunteer participants were thanked for their availability, and contact references were given for any questions about the purpose of the research. Also, the online system did not guarantee the possibility of saving the questionnaire without having definitively concluded it.

For our sample, the results indicate that the instruments used has really good internal consistency. Specifically, α = 0.82 was for FCV19S, α = 0.92 for COVID-19-PTSD test, α = 0.97 for POMS, and α = 0.89 for the BMI-HSS.

#### Fear of COVID-19 Scale

The Fear of COVID-19 scale (FCV19S) (18) represents a standardized tool in assessing the generalized fear of COVID-19 among individuals, fear often associated with the transmission speed, and the high mortality rate related to the virus. The scale showed good reliability (α = 0.87) and is a one-dimensional questionnaire composed of seven items (e.g., “I'm very afraid of coronavirus-19”; “It makes me uncomfortable to think about coronavirus-19”; “I can't sleep because I worry about getting coronavirus-19”), with a five-point response scale (1 = strongly disagree to 5 = strongly agree), which assesses fear of COVID-19 and its consequences. The score is obtained by adding the scores to the questions.

#### COVID-19—Posttraumatic Stress Disorder

A section of the questionnaire used the COVID-19—PTSD (1, 19) to investigate posttraumatic stress disorder (F43.10). This questionnaire includes 19 items (e.g., “Having repeated, disturbing and unwanted thoughts related to this stressful experience,” “To have difficulty in falling asleep”), requiring a response on a five-point Likert scale, from 0 (not at all) to 4 (extremely), and is developed, thanks to the modification of the PCL-5 (22) in order to focus the attention on a prolonged and current stressor. A COVID-19—PTSD cutoff score of 26 was deemed to correctly categorize a participant as having or not having significant PTSD symptoms.

The COVID-19—PTSD demonstrated a good internal consistency (Cronbach's α = 0.94) and a robust convergent validity.

#### Profile of Mood States

The Profile of Mood States (POMS) scale is a widespread psychological instrument used to measure mood and identify problematic affective states. The scale, developed by McNair et al. (20) is composed of a list of adjectives that measure six aspects or scales of emotions. The POMS scale showed good reliability (α = 0.85) and consists of a questionnaire of 58 adjectives (e.g., “Tense,” “Energetic,” “Fatigued”), is particularly useful in evaluating subjects with stress disorders, and is structured on the basis of six mood states: tension–anxiety (T), which describes an increase in somatic tension that may not be observable from the outside or may concern visible psychomotor manifestations; depression (D), which indicates a state of depression accompanied by a sense of personal inadequacy, the uselessness of effort, a sense of emotional isolation, melancholy, and guilt; aggression–anger (A), which describes anger and dislike toward others; vigor–activity (V), a positive factor including exuberance, energy, euphoria, and optimism; tiredness–indolence (TI), which represents boredom, low energy, and physical fatigue; and confusion (C), characterized by a sense of disturbance and linked to the organization–disorganization dimension, anxiety, and the feeling of cognitive inefficiency. The intensity of the mood is measured on a five-point Likert scale (0 = not at all, 1 = a little bit, 2 = moderately, 3 = quite a bit, and 4 = extremely). Total scoring for the scale [Total Mood Disturbance (TMD)] can be calculated by adding the scores for tension, depression, anger, tiredness, confusion, and then subtracting the score for vigor.

#### Maslach Burnout Inventory—Human Services Survey

Burnout is a syndrome of high emotional exhaustion and high depersonalization in the presence of a lack of personal accomplishment. The Maslach Burnout Inventory—Human Services Survey (MBI-HSS) is a questionnaire of 22 items, each of which with 7 degrees of response on the Likert scale (0 = never, 1 = a few times a year or less, 2 = once a month or less, 3 = a few times a month, 4 = once a week, 5 = a few times a week, 6 = every day). This questionnaire was designed for professionals in human service employees and is appropriate for respondents working in a diverse array of occupations, including nurses, and other fields focused on helping people live better lives by offering guidance, preventing harm, and ameliorating physical, emotional, or cognitive problems. The questionnaire was developed by Maslach and Jackson (23) and investigates three different subscales: emotional exhaustion (EE—nine items—e.g., “I feel burned out from my work”), depersonalization (DP—five items—e.g., “I worry that this job is hardening me emotionally”), and personal accomplishment (PA—eight items—e.g., “In my work, I deal with emotional problems very calmly”). Scales are scored such that higher scores indicate more of each construct. Higher scores on the EE and DP subscales indicate a higher burnout symptom burden; lower scores on the PA subscale indicate a higher burnout symptom burden (21). The reliability of all items measured by Cronbach's index was 0.80 for the Italian version used (24). This scale was not considered for unemployed respondents.

### Data Analysis

The SPSS version no. 26 was used for the statistical analyses. We analyzed the data using parametric techniques when the data satisfied the assumptions of normality of the distribution, i.e., Student's *t* and discriminant analysis for detecting significant groups differences, Pearson's *r*, and multiple regression for correlational analyses. In analyzing the compared groups based on criterial variables, we used chi-square statistic.

## Results

The fear of COVID is not significantly different in the two groups considered in the study: in healthcare professionals, mean 15.08, *SD* 4.95; in the controls, mean 14.66, *SD* 4.82 (*t* = 0.63, df = 212, *p* = 0.53).

Instead, in the two groups, both POMS factors and the Total Mood Disturbance (TMD) significant differences have been found, with higher scores in the controls. However, the factors vigor and tiredness are not significant (Table 1).

**Table 1:** Means, *t*-test, and *p*-value for the comparison between groups in the factors and total score of Profile of Mood States (POMS).

**POMS**	**Group**	**Mean**	**SD**	***t***	***p*** **(df = 212)**
T—Tension	Healthcare workers (HCWs)	11.77	7.78	−2.03	0.04^*^
	Control	14.08	8.86		
D—Depression	HCWs	15.50	11.90	−2.34	0.02^*^
	Control	19.79	14.83		
A—Anger	HCWs	11.51	9.98	−3.64	<0.01^**^
	Control	17.07	12.25		
V—Vigor	HCWs	17.90	5.49	1.32	0.19
	Control	16.73	7.34		
TI—Tiredness	HCWs	10.80	6.05	−1.46	0.14
	Control	12.09	6.82		
C—Confusion	HCWs	8.29	5.81	−3.47	<0.01^**^
	Control	11.15	6.26		
TMD—Total Mood Disturbance	HCWs	39.97	40.04	−2.87	<0.01^**^
	Control	57.46	48.79		

** *The symbol indicates the value of p < 0.01 and symbol*

* *indicates the value of p < 0.05*.

Table 2 shows that in two out of three MBI-HSS factors, the scores are significantly higher in the healthcare professionals group than in the control group (excluding the not employee respondents).

**Table 2:** Mean, *t*-test, and *p*-value for the comparison between groups in the Maslach Burnout Inventory—Human Services Survey (MBI-HSS) (only working respondents were considered: *N* = 96).

**MBI-HSS**	**Group**	**Mean**	**SD**	***t***	***p*** **(df = 201)**
EE—Emotional Exhaustion	HCWs	19.81	10.10	1.96	0.05^*^
	Control	16.68	12.72		
DP—Depersonalization	HCWs	12.63	5.39	6.42	<0.01^**^
	Control	7.06	6.93		
PA—Personal Accomplishment	HCWs	28.18	7.64	1.58	0.12
	Control	26.27	9.57		

** The symbol indicates the value of p < 0.01 and symbol ^**^indicates the value of p < 0.05.

Emotional exhaustion and depersonalization are higher in the professionals, while personal accomplishment at work is higher too, but not at a significant level.

PTSD values are high in both groups, higher—but not in a significant level—in healthcare professionals (*n* = 107, mean = 26.18, *SD* = 14.60) compared vs. controls, excluding nonprofessional participants (*n* = 85, mean = 25.75, *SD* = 14.65; *t* = 0.20, *p* = 0.84).

Also, considering the participants with COVID-19—PTSD scores higher than the cutoff (25), the differences between the two groups are not significant: 52.34% (*N* = 54) among health professionals vs. 47.06% (*N* = 52) of controls (χ^2^ = 0.53, *p* = 0.47).

Given that the two groups are not significantly different in PTSD scores, we have computed the correlations between the level of stress and the other variables in the whole sample. In the study, all the correlations are highly significant, except for vigor and personal accomplishment at work (Table 3).

**Table 3:** Pearson correlation of COVID-19—Posttraumatic Stress Disorder (PTSD) with Fear of COVID-19 scale (FCV19S), POMS—TMD and subscale, and MBI-HSS scores.

	**COVID-19-PTSD**
FCV19S—Fear of COVID-19	0.76^**^
POMS—T (Tension)	0.68^**^
POMS—D (Depression)	0.59^**^
POMS—A (Anger)	0.57^**^
POMS—V (Vigor)	−0.16
POMS—S (Tiredness)	0.67^**^
POMS—C (Confusion)	0.50^**^
POMS—TMD (Total Mood Disturbance)	0.63^**^
EE—Emotional Exhaustion	0.44^**^
DP—Depersonalization	0.36^**^
PA—Personal Accomplishment	0.07

** *The symbol indicates the value of p < 0.01*.

Posttraumatic stress is significantly correlated with the fear of COVID-19 and other negative emotions (mostly with tension, tiredness, anger, and confusion). Also, emotional exhaustion and depersonalization at work are connected with general stress due to the pandemic event.

To better differentiate the two groups based on the scores in the standardized tests, a discriminant analysis was performed (Table 4), using a predictor of the POMS—Total Mood Disturbance score, the Fear of COVID-19, and the three factors of MBI-HSS scores.

**Table 4:** Results of discriminant analysis (Wilks' Λ = 0.67, χ^2^ = 20.05, *p* < 0.001).

**Variables**	**F-to-remove**	**Coefficient**
POMS—TMD (Total Mood Disturbance)	24.79	0.49
DP—Depersonalization	54.88	0.34
PA—Personal Accomplishment	4.34	0.74
FCV19S—Fear of COVID-19	1.51	0.81
EE—Emotional Exhaustion	2.16	0.29

Total mood disturbance and depersonalization are the most discriminating variables.

The classification results confirm that the discriminant function based on the test variables can distinguish the two groups with a percent of correct of a medium-high level (77%), more for controls (80%) than for healthcare professionals (74%).

After analyzing the difference between groups, other analyses were addressed to the study of the relations within the target group, i.e., the healthcare professionals. The correlations among the MBI-HSS and POMS factors in healthcare professionals are shown in the Table 5. Emotional exhaustion and depersonalization are correlated with all POMS factors except with vigor. Also, the personal accomplishment factor (scored in the positive direction) correlates only with vigor (also positive factor), not significantly with other variables.

**Table 5:** Person correlations among the MBI-HSS and POMS factors in healthcare professionals' group.

**MBI—Factors**	**POMS—Factors**					
	**Tension**	**Depression**	**Anger**	**Vigor**	**Tiredness**	**Confusion**
EE—Emotional Exhaustion	0.64^**^	0.74^**^	0.62^**^	−0.17	0.75^**^	0.64^**^
DP—Depersonalization	0.41^**^	0.52^**^	0.49^**^	−0.20	0.60^**^	0.40^**^
PA—Personal Accomplishment	0.19	0.16	0.10	0.37^**^	0.21	0.04

** *The symbol indicates the value of p < 0.01*.

Moreover, fear of COVID measured by FCV19S test significantly (*p* < 0.01) correlates with POMS—Total Mood Disturbance (0.49), with factors Tension (0.52), Depression (0.47), Anger (0.33), Tiredness (0.57), Confusion (0.38), and not with Vigor (−0.16).

Table 6 shows the results of a series of multiple regression analyses performed in healthcare professional samples separately for the three variables of the MBI-HSS.

**Table 6:** Multiple regressions for the three variables of the MBI-HSS in healthcare professional samples.

**Predictors:**	**EE Emotional exhaustion**	**DEP Depersonalization**	**PA Personal accomplishment**
	**Std. coeff**.	**Std. coeff**.	**Std. coeff**.
	***r***^**2**^ **= 0.61**	***r***^**2**^ **= 0.44**	***r***^**2**^ **= 0.28**
FCV19S—Fear of COVID-19	0.07	0.00	−0.03
POMS—T (Tension)	−0.21	−0.45^*^	0.11
POMS—D (Depression)	0.48^**^	0.10	0.19
POMS—A (Anger)	−0.07	0.44^*^	−0.49^*^
POMS—V (Vigor)	0.04	−0.12	0.49^***^
POMS—S (Tiredness)	0.45^***^	0.74^***^	0.48^*^
POMS—C (Confusion)	0.10	−0.28	−0.03

*Predictors are the scores on the Fear of COVID-19 scale and POMS subscales*.

The symbol ***indicates the value of p < 0.001, the symbol

** *indicates the value of p < 0.01, and symbol*

* *indicates the value of p < 0.05*.

Results demonstrate that depression and tiredness are the best predictors of emotional exhaustion; tiredness is the best predictor also of depersonalization, together with anger and negation of tension. Vigor and tiredness, conjointly with reduced anger, predict personal accomplishment in healthcare professionals.

## Discussion and Conclusions

Anxiety, depression, burnout, and suicide risk among healthcare workers (HCWs) were considered as critical health issues even before the COVID-19 pandemic (26). However, the coronavirus disease-19 (COVID-19) has brought about a period of world emergency and highlighted the need to focus on the impact caused by the pandemic situation both in the subjects directly involved in the management of this emergency and in the general population. Recent cross-sectional studies reported that increased workload and burnout were especially pronounced among frontline HCWs who volunteered as members of the COVID-19 outbreak response team (25, 27–30). Previous studies of frontline health workers during the SARS and Ebola outbreaks showed that frontline workers suffer significant risks of burnout, anxiety, and PTSD (31–33). However, the psychological suffering that follows exposure to a traumatic and stressful event is highly variable. For this reason, it is not uncommon for the clinical picture to include some combinations of symptoms (e.g., anhedonia, dysphoria, anger, and dissociation) with the presence or absence of anxiety and fear. A recent systematic review (34) showed that 29 studies reported the prevalence of mental health disorders in HCWs. Specifically, the percentage of healthcare workers with anxiety ranged from 9 to 90% with a median of 24%, while the percentage with depression ranged from 5 to 51%, with a median of 21%.

This cross-sectional online study intended to examine the prevalence of PTSD symptomatology and the emotional impact in Italian healthcare workers and the general population during the phases immediately following the possibility of administering vaccines and medical treatment for COVID-19 (over 2 months, from March to May 2021).

The results of the study have indicated that both the groups of our sample show a high level of posttraumatic stress derived from working during a pandemic, with nearly half of the professionals exceeding the cutoff (>26) in accordance with the Italian standardization of the COVID-19—PTSD test (1, 19). Comparing the two groups, we found that healthcare professionals have a consistent perception of stress, but not at a level significantly higher than other categories of workers. However, probably as a result of their specific training and supervision, they showed less emotional disturbance than the control sample, as they are familiar with, and capable of, dealing with more stress, have better anxiety management skills, and display lower values of depressive disorder and mental confusion. Instead, the healthcare professionals showed a condition of emotional exhaustion and depersonalization, which is common in the burnout syndrome. These symptoms, in the group of healthcare professions, are predicted by specific emotional variables: e.g., Tiredness together with Depression due to Emotional Exhaustion, Tension, Anger, and Depersonalization.

It is, therefore, recommended that the HCWs are provided with a safe and secure environment that promotes their psychological wellbeing to facilitate adequate service delivery during the COVID-19 pandemic and future events of disease outbreak (35). As suggested by Tucci et al. (32), whereas HCWs are not sufficiently capable of managing their individual health while caring for other ill persons, this supports the need for national and local healthcare agencies to place a premium on the psychological and mental health status of HCWs (35). Intervening professionally on the outcomes found on the emotional sphere in times of crisis, as the epidemiological situation in the grip of the COVID-19 pandemic demonstrates, means learning to manage emergency situations and also dealing with them on the psychic side.

Some limitations of this study need to be acknowledged. First, the number of healthcare professionals and controls were not high enough to make differentiation among the jobs. Second, as the sample was not representative of the healthcare workers population, the study should be considered a correlational one. Furthermore, the use of self-report instruments and the lack of data about COVID-19 infection or other variables related to the pandemic (death of a loved one, etc.) may be considered limitations.

In conclusion, the results obtained are useful in understanding the determinants of the emotional involvement of healthcare professions and the risk of burnout syndrome and, therefore, for planning activities and support paths for these workers who are particularly at risk during prolonged and pervasive crises, such as the pandemic. As suggested by Chirico et al. (35), social activities, such as sharing one's experience with colleagues and family members, would help reduce subthreshold syndromes before they evolve to complex conditions. Scientific literature confirms the positive effect of practicing oriental disciplines as Judo, Tai Chi, yoga, or meditation on health and self-control to recover our balance (36). Furthermore, psychological support interventions for healthcare workers should not be limited to a set period of time (e.g., lockdown), but should be constantly monitored and guaranteed regardless of the crisis events. However, further research could be needed to comprehend their cost effectiveness for individuals and health organizations and their sustainability over time.

## Data Availability Statement

The raw data supporting the conclusions of this article will be made available by the authors, without undue reservation.

## Ethics Statement

The studies involving human participants were reviewed and approved by Department of Educational Sciences of the University of Catania (Italy). The patients/participants provided their written informed consent to participate in this study.

## Author Contributions

CDe, DC, and SD: conceptualization and methodology. DC: validation. DC and SD: formal analysis. RD'A and EL: investigation. SD: data curation and supervision. DC and RD'A: writing—original draft preparation. CDe, DC, CDi, and SD: writing—review and editing. CDe: funding acquisition. All authors contributed to the article and approved the submitted version.

## Funding

This research has been fully supported by the project PIACERI 2020 (PIAno di inCEntivi per la RIcerca di Ateneo) of the Department of Educational Sciences, University of Catania (Italy). Project: Self-care, care of the world. The impact of the environmental crisis on the physical (soma) and moral (psyche) of man.

## Conflict of Interest

The authors declare that the research was conducted in the absence of any commercial or financial relationships that could be construed as a potential conflict of interest.

## Publisher's Note

All claims expressed in this article are solely those of the authors and do not necessarily represent those of their affiliated organizations, or those of the publisher, the editors and the reviewers. Any product that may be evaluated in this article, or claim that may be made by its manufacturer, is not guaranteed or endorsed by the publisher.
